# Two new and one newly recorded species of Gracillariidae from China (Lepidoptera)

**DOI:** 10.3897/zookeys.559.6812

**Published:** 2016-02-03

**Authors:** Haiyan Bai, Jiasheng Xu, Xiaohua Dai

**Affiliations:** 1 Department of Bioscience and Biotechnology, Changzhi College, No. 73, East street north of the city, Changzhi, 046011 Shanxi Province, P. R. China; 2 School of Life and Environmental Science, Gannan Normal University, South of College Road, Economic-Technological Development Area, Ganzhou, 341000 Jiangxi Province, P. R. China

**Keywords:** China, Gracillariidae, new species, new record, taxonomy

## Abstract

The paper presents four Chinese species belonging to the genera *Metriochroa* Busck, *Eumetriochroa* Kumata, and *Gibbovalva* Kumata & Kuroko (Lepidoptera, Gracillariidae), including two new species: *Metriochroa alboannulata* Bai, **sp. n.** and *Gibbovalva clavata* Bai, **sp. n.** *Eumetriochroa hiranoi* Kumata, 1998, is newly recorded from China. Photographs of adults and figures of the genital structures are provided, along with keys to the Chinese species of *Metriochroa*, *Eumetriochroa*, and *Gibbovalva*.

## Introduction

*Eumetriochroa* Kumata, 1998 and *Metriochroa* Busck, 1900 are small genera of Gracillariidae Oecophyllembiinae (Kobayashi et al. 2013; De Prins and De Prins 2015). The genus *Eumetriochroa* contained four new species worldwide when it was erected (Kumata, 1998), namely *Eumetriochroa hederae* Kumata, 1998, *Eumetriochroa hiranoi* Kumata, 1998, *Eumetriochroa kalopanacis* Kumata, 1998 and *Eumetriochroa miyatai* Kumata, 1998. A new species, *Eumetriochroa araliella* Kobayashi, Huang & Hirowatari, 2013, was subsequently added to the genus (Kobayashi et al. 2013). Accordingly, five species are currently recognized in *Eumetriochroa* worldwide, all of them originally recorded from Japan. Larvae are leaf-miners on Aquifoliaceae, Araliaceae, and Styracaceae. To date eleven plant species in seven genera have been recorded as host plants of *Eumetriochroa* (Kumata 1998; Kobayashi et al. 2011, 2013; De Prins and De Prins 2015). Prior to this study, *Eumetriochroa* was represented in China by only one species, *Eumetriochroa hederae*, firstly reported there by Kobayashi et al. (2011).

The genus *Metriochroa* contains twelve described species worldwide. There are seven species in the Afrotropical region, three in the Palearctic region, and one each in the Oriental and Nearctic regions. *Metriochroa* was not recorded in China until *Metriochroa symplocosella* Kobayashi, Huang & Hirowatari, 2013 was described on the basis of Chinese material (Kobayashi et al. 2013). A total of twenty plant species in twelve genera of six families are known as host plants of *Metriochroa*. Eleven species in five genera of the family Oleaceae serve as the most common host plants for the larvae of *Metriochroa* (Kumata 1998; Kobayashi et al. 2013; De Prins and De Prins 2015).

The genus *Gibbovalva* Kumata & Kuroko, 1988 is one of the smallest genera of the subfamily Gracillariinae, and is represented by eight species worldwide. The majority of them (five) occur in the Palearctic and Oriental regions, the remaining two species were recorded from the Australasian region and one from the Afrotropical region. Host plants of *Gibbovalva* comprise thirty-four species in ten genera under four families, the majority of which (twenty species in six genera) belong to the family Lauraceae, followed by the Magnoliaceae (ten species in two genera), the Typhaceae (three species in one genus), and the Apocynaceae (one species) (Kumata et al. 1988; Bai and Li 2008; Bai et al. 2009; Triberti and Jaworski 2014, De Prins and De Prins 2015). Six species of *Gibbovalva* were recorded in China, mainly distributed in Guangdong, Guangxi, Hainan, Fujian, Guizhou, Zhejiang, Anhui, Yunnan, Hunan, Liaoning and Hong Kong (Bai and Li 2008).

Of the four gracillarid moth species treated in the present paper, *Eumetriochroa hiranoi* is newly recorded from China, and *Metriochroa alboannulata* sp. n. and *Gibbovalva clavata* sp. n. are new to science.

## Methods

All adult specimens were obtained after by rearing from immature stages. Adult external morphology was examined by using a Leica M-205C stereomicroscope, and photographs were taken with a Leica DFC-450 digital camera connected to a Leica M-205C stereomicroscope. Genitalia were prepared following the methods of Li and Zheng (1996). Dissections of genitalia were conducted under an Olympus SZX-7 stereomicroscope. Genital morphology was examined with an Olympus BX-53 microscope, and the illustrations were prepared by using an Olympus DP-26 digital camera connected to the Olympus BX-53 microscope. Terminology follows Kumata (1998) and Kumata et al. (1988).

All specimens studied are deposited in the Insect Collection, Department of Bioscience and Biotechnology, Changzhi College, Changzhi, Shanxi, China (ICCC).

## Taxonomy

### Eumetriochroa

Taxon classificationAnimaliaLepidopteraGracillariidae

Kumata, 1998

Eumetriochroa Kumata, 1998, *Insecta Matsumurana* (N.S.) 54: 83.

#### Type species.

*Eumetriochroa hederae* Kumata, 1998.

#### References.

Kumata (1998: 85, figs 1, 2A, 12A, B, 14A, 17, 18A, 22A, 24A, B).

#### Key to the Chinese species of *Eumetriochroa*

**Table:** 

1	Forewing snow white, with five ochreous brown fasciae; (♂) valva with a trapezoid lobe on disc, vesica with a weakly sclerotized tubular part; (♀) signum blade-shaped, with a laterally elongated, triangular basal plate	***Eumetriochroa hiranoi* Kumata, 1998**
–	Forewing ochreous brown, with five white fasciae; (♂) valva with a finger shaped process basally, vesica with a cornutus which has three to four transverse dentils; (♀) signum thorn-shaped, with a small triangular basal plate	***Eumetriochroa hederae* Kumata, 1998**

### Eumetriochroa hiranoi

Taxon classificationAnimaliaLepidopteraGracillariidae

Kumata, 1998

Figs 1 , 5 , 9

Eumetriochroa hiranoi Kumata, 1998, *Insecta Matsumurana* (N.S.) 54: 96.

#### References.

De Prins & De Prins (2005: 185), Kobayashi et al. (2013: 119).

#### Adult

**Figures 1–4.**
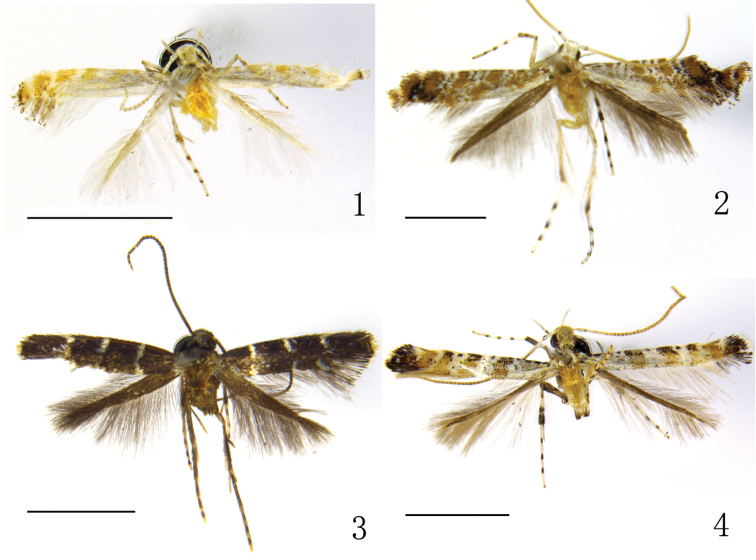
Adults. **1** *Eumetriochroa hiranoi* Kumata **2** *Eumetriochroa hederae* Kumata **3** *Metriochroa alboannulata* Bai, sp. n. **4** *Gibbovalva clavata* Bai, sp. n. Scale bar 2000 μm.

(Fig. 1). Wing expanse 4.7~5.1 mm.

#### Material examined.

2♂♂, 2♀♀, China. Feng Shan, Ganzhou, Jiangxi Province, 8 September 2012, leg. Jiasheng Xu and Chengqing Liao; genitalia slide Nos B13087, B13088, B13089, B13090; all in ICCC.

#### Host plant.

Styracaceae: *Styrax japonicus* Siebold & Zucc. (Kumata, 1998).

#### Distribution.

China (Jiangxi), Japan.

#### Remarks.

**Figures 5–8.**
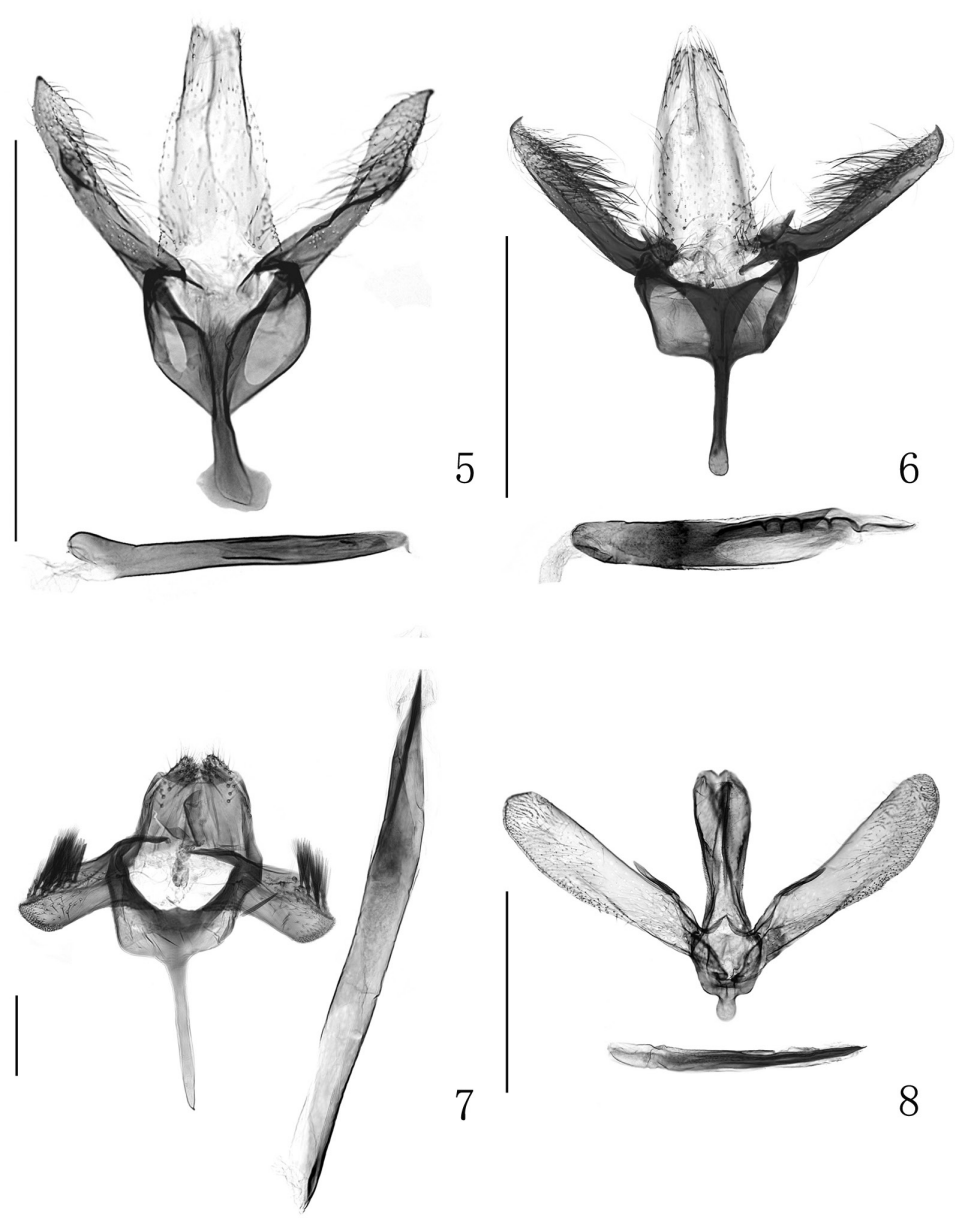
Male genitalia. **5** *Eumetriochroa hiranoi* Kumata **6** *Eumetriochroa hederae* Kumata **7** *Metriochroa alboannulata* Bai sp. n. **8** *Gibbovalva clavata* Bai, sp. n. Scale bar 500 μm.

**Figures 9–10.**
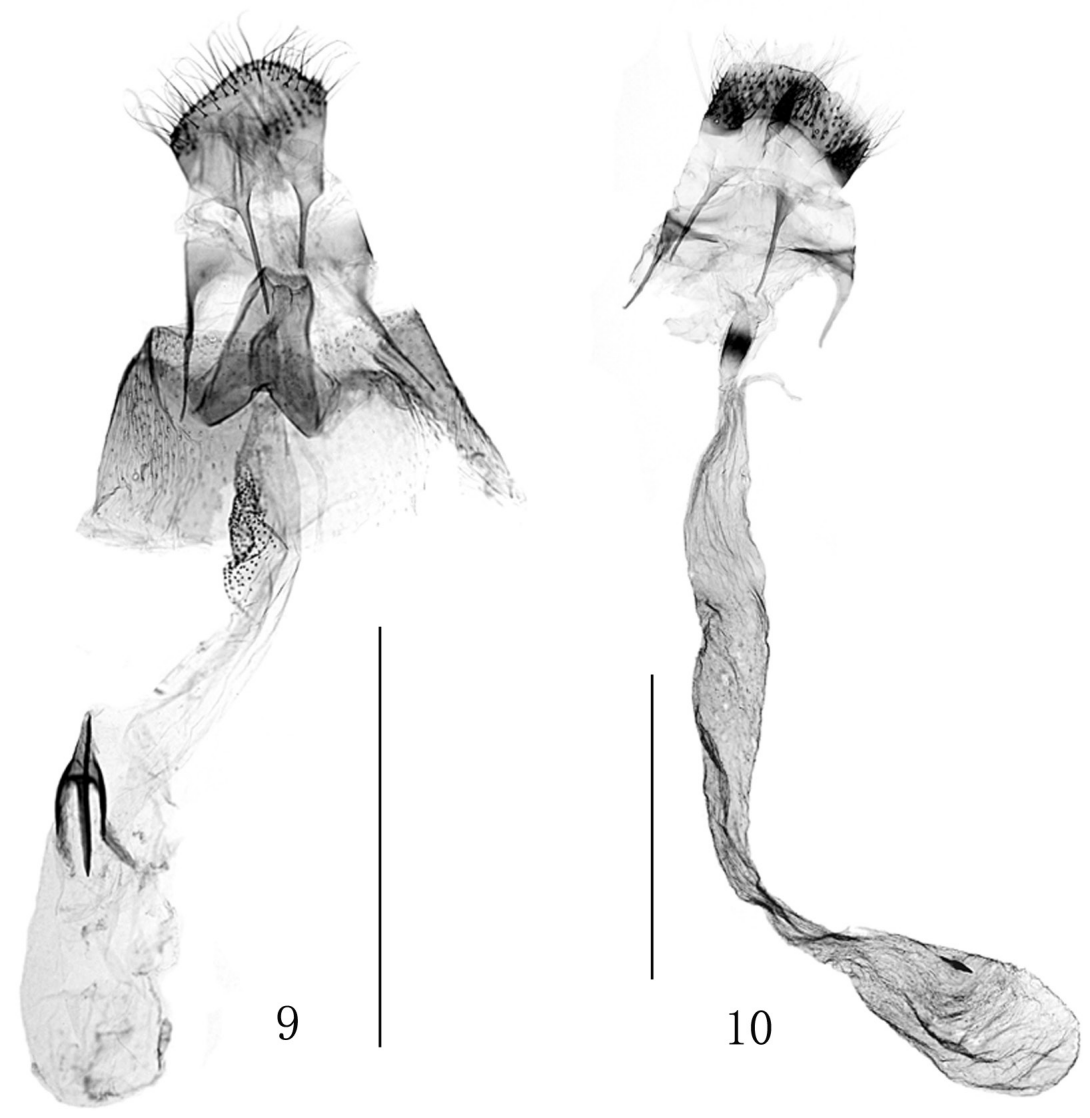
Female genitalia. **9** *Eumetriochroa hiranoi* Kumata **10** *Eumetriochroa hederae* Kumata. Scale bar 500 μm.

In all four specimens examined the fore wing markings were poorly preserved, but the genital characters (Figs 5, 9) well agree with the description given by Kumata (1998). First record from China.

### Eumetriochroa hederae

Taxon classificationAnimaliaLepidopteraGracillariidae

Kumata, 1998

Figs 2 , 6 , 10

Eumetriochroa hederae Kumata, 1998, *Insecta Matsumurana*. (N.S.) 54: 85.

#### References.

De Prins and De Prins (2005: 185), Kobayashi et al. (2011: 28).

#### Adult

(Fig. 2). Wing expanse 8.1~8.7 mm.

#### Material examined.

China. 1♂, Daqiutian, Jiulian Mountain, Jiangxi Province, 18 January 2013, leg. Xiaohua Dai; 2♀♀, Yangling National Forest Park, Chongyi County, Jiangxi Province, 700 m, 10 March 2012, leg. Jinshui Liang; genitalia slide Nos B12011, B12012, B13057; all in ICCC.

#### Host plants.

Araliaceae: *Hedera sinensis* (Tobler) Hand.-Mazz.; *Hedera rhombea* (Miq.) Bean (Kumata 1998; Kobayashi et al. 2011).

#### Distribution.

China (Hunan, Jiangxi), Japan.

#### Remarks.

Specimens from China do not fully agree with the original description (Kumata 1998), especially in fore wing markings. Their fore wing has a white stripe situated between the third and fourth fasciae which extends from the dorsal edge of the third fascia towards costa to the middle of the fourth fascia. This character was not recorded by Kumata (1998) in the original description based on Japanese specimens. In addition, instead of the fourth fascia as described by Kumata, it is the apex of the fifth fascia which is edged with remarkable darker spots. However, the structures of the male (Fig. 6) and the female genitalia (Fig. 10) are in accordance with the original description, which provides us confidence to assign the specimens reared in China to this species.

### Metriochroa

Taxon classificationAnimaliaLepidopteraGracillariidae

Busck, 1900

Metriochroa Busck, 1900, *Proceedings of the United States National Museum* 23: 244.

#### Type species.

*Metriochroa psychotriella* Busck, 1900.

#### References.

Busck (1900: 245, pl. 1, fig. 13).

#### Key to the Chinese species of *Metriochroa*

**Table:** 

1	Fore wing fuscous, with three white fasciae; (♂) vesica with a clavate cornutus	***Metriochroa alboannulata* sp. n.**
–	Fore wing pure to ochreous white, with three fuscous obscure specks; (♂) vesica with a bundle of spines at the middle	***Metriochroa symplocosella* Kobayashi, Huang & Hirowatari, 2013**

### Metriochroa alboannulata

Taxon classificationAnimaliaLepidopteraGracillariidae

Bai sp. n.

http://zoobank.org/40AAAD7F-773E-4932-9C33-BD34A83D068D

Figs 3 , 7 , 11

#### Diagnosis.

**Figure 11.**
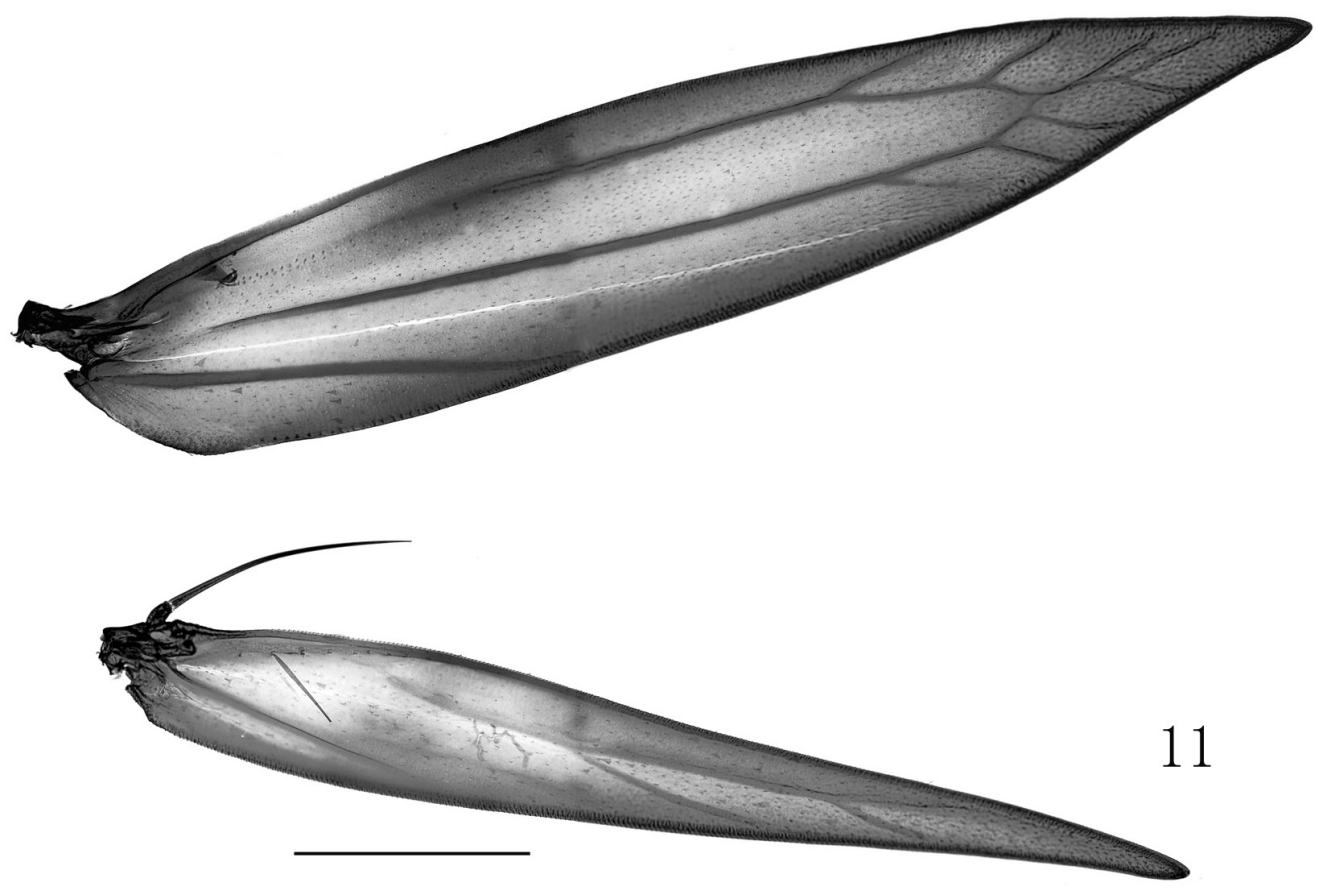
Wing venation of *Metriochroa alboannulata* Bai, sp. n. Scale bar 500 μm.

The new species is a member of *Metriochroa* by the characteristics of venation and male genitalia. Fore wing of *Metriochroa alboannulata* sp. n. has nine veins (Fig. 11), M_3_ and CuA are absent, M_1_ is stalked with R_5_, R_4_ is connate or shortly stalked with stalk of M_1_ and R_5_; the valva is covered with partite scales.

Flagellum of *Metriochroa alboannulata* sp. n. has six white rings on distal part. Forewing has two silvery white fasciae: one placed at the basal 1/4 and is slightly outwardly angulate on wing fold, the other situated preapically; forewing possesses white costal and dorsal specks, two of them at the middle, and opposite each other, and one near the tornus. Valva is divided into dorsal and ventral portions by a sclerotized ridge, the former shorter than the ventral one. Aedeagus is tubular, and with a clavate cornutus on vesica.

Forewing markings of *Metriochroa* vary notably. *Metriochroa alboannulata* sp. n. is similar to *Metriochroa argyrocelis* Vári, 1961 and *Metriochroa celidota* Bradley, 1965 in forewing with obvious white or silvery white markings. These characteristics easily distinguish these species from other members of the genus.

*Metriochroa alboannulata* is close to *Metriochroa celidota* in forewing with two silvery white fasciae, especially as the first fascia is present at the basal 1/4 in both species. However, in *Metriochroa alboannulata* the first fascia is evident and joins with dorsum, and the second fascia is closer to the apex of forewing than in *Metriochroa celidota*; in addition, *Metriochroa alboannulata* has a silvery white speck near tornus, which does not occur in *Metriochroa celidota*.

Both *Metriochroa alboannulata* and the female of *Metriochroa argyrocelis* (forewing markings of male *Metriochroa argyroscelis* are clearly dissimilar from those of *Metriochroa alboannulata*) have a silvery white fascia at the basal 1/4 of forewing, and a silvery white speck near tornus, but they differ in the following characteristics: in *Metriochroa alboannulata*, the fascia is of uniform width, and is narrower than that of *Metriochroa argyrocelis*, in which it gradually widens towards dorsum; in addition, in place of the fascia near the apex of forewing and the silvery white bar-shaped specks at the middle of costa and dorsum present in *Metriochroa alboannulata*, *Metriochroa argyrocelis* has two silvery white specks at the middle and basal 3/4 of costa, respectively.

#### Description.

Adult (Fig. 3). Wingspan 6.5–7.5 mm. Head fuscous with metallic luster. Antenna fuscous, flagellum with six white rings on distal part. Labial palpus whitish-yellow, with the outer side of second and third segments fuscous. Thorax, tegula, and fore wing fuscous. Fore wing shining with purple; two silvery white fasciae present, first fascia at the basal 1/4, and slightly outwardly angulate on wing fold, second fascia at subapex and outwardly oblique; costa and dorsum with an outwardly oblique bar-shaped silvery white speck each at the middle, costal speck longer than the dorsal one; dorsum with a silvery white speck near tornus; cilia grayish-brown, those on termen with median and apical fringe lines of black spots, which run parallel with termen. Hindwing and its cilia fuscous. Legs fuscous. External surface of profemur and mesofemur, internal surface of metafemur ochreous white; protibia basally, mesotibia and extremities of metatibia ochreous white; both ends of first tarsomeres, apical tarsomeres and the apex of other tarsomeres white. Abdomen dorsally fuscous, ventrally ochreous white, anterior margin of each sternite fuscous.

Male genitalia (Fig. 7). Tegumen *ca.* 100 μm in length, with widely rounded apex. Tuba analis bilobed apically, with setae on each lobe. Vinculum Y-shaped; saccus *ca.* 180 μm in length, clavate, with pointed apex. Valva *ca.* 160 μm in length, about three times as long as wide; inner surface with a sclerotized longitudinal ridge which divides the valva into dorsal and ventral portions; dorsal portion slightly shorter than ventral one, with obliquely truncated apex, and covered with a group of partite scales on its distal part; ventral portion with spine-like setae on its rounded apex. Aedeagus tubular, *ca.* 700 μm long, obliquely truncated along apical 2/7, pointed apically; vesica with a clavate cornutus, which is approximately 160 μm long.

Female. Unknown.

#### Type material.

Holotype ♂. China. Wuzhifeng, Shangyou County, Jiangxi Province, 2 January 2013, leg. Chengqing Liao; genitalia slide No. B13051, in ICCC. Paratypes 2♂♂. China, with same data as holotype; genitalia slide Nos B13050, BX15001, in ICCC.

#### Etymology.

The specific name is composed of “*albus*” and “*annulatus*”, meaning “with white ring”, referring to the flagellum of antenna with white rings on its distal part.

#### Distribution.

China (Jiangxi).

### Gibbovalva

Taxon classificationAnimaliaLepidopteraGracillariidae

Kumata & Kuroko, 1988

Gibbovalva Kumata & Kuroko, 1988, In: Kumata, Kuroko and Ermolaev, 1988, *Insecta Matsumurana* (N.S.) 40: 3

#### Type species.

*Gracilaria* (sic) *quadrifasciata* Stainton, 1862.

#### Reference.

Stainton (1862: 295, pl. 10, fig. 5).

#### Key to the Chinese species of *Gibbovalva*

**Table:** 

1	Forewing with five white or ochreous yellow fasciae	**2**
–	Forewing with four white fasciae	**5**
2	Forewing fuscous with ochreous yellow fasciae	***Gibbovalva civica* (Meyrick, 1914)**
–	Forewing ochreous yellow with white fasciae	**3**
3	Antenna with flagellum white in several basal segments; aedeagus without cornutus	***Gibbovalva urbana* (Meyrick, 1908)**
–	Antenna with flagellum ochreous yellow to ochreous brown; aedeagus with cornutus	**4**
4	Forewing with fifth fascia intercalated by a black narrow line in centre; aedeagus with a flap like process at basal 1/3	***Gibbovalva kobusi* Kumata & Kuroko, 1988**
–	Fifth fascia without the aforementioned characteristic; flap like process of aedeagus absent	***Gibbovalva magnoliae* Kumata & Kuroko, 1988**
5	Forewing fuscous, white fasciae with large evident fuscous spots, third fascia interrupted by ground color	***Gibbovalva quadrifasciata* (Stainton, 1862)**
–	Forewing ochreous yellow or distal 2/3 ochreous, fuscous spots in white fasciae obscure or absent, fasciae uninterrupted	**6**
6	First fascia at base of forewing; three basal white fasciae wider than the fourth one, and almost equal in width; forewing with a black speck on dorsum between third and fourth fasciae, and a white speck above the black one	***Gibbovalva singularis* Bai & Li, 2008**
–	First fascia nearly at the middle of forewing, the first and the second fasciae wider than the third and the fourth fasciae; space between third and fourth fasciae without spots	***Gibbovalva clavata* sp. n.**

### Gibbovalva clavata

Taxon classificationAnimaliaLepidopteraGracillariidae

Bai sp. n.

http://zoobank.org/C81BC6DD-CFDF-4DC9-85BF-EEA4FFCC83EF

Figs 4 , 8

#### Diagnosis.

A new species of *Gibbovalva*, with antennal scape bearing a ventral flap and valva with a costal process as for other members of the genus, with which it also shares the fore wing markings and characteristics of vinculum and saccus. The basal 1/3 of the forewing of *Gibbovalva clavata* sp. n. is white in ground color and has four black costal specks; the distal 2/3 is ochreous yellow in ground color and has four white fasciae. The valva is blade-shaped, the costa possesses a clavate process at the basal 1/6; saccus is thumb-shaped with rounded apex; the aedeagus does not have a flap-like process, and its thorn-like cornuti are arranged in rows from basal 1/3 to subapex.

*Gibbovalva clavata* sp. n. is similar to *Gibbovalva magnoliae* and *Gibbovalva tricuneatella* in the aedeagus lacking a flap-like process, but it is distinguishable by the forewing markings. In *Gibbovalva clavata*, basal 1/3 of forewing is white with four black costal specks; apical 2/3 of forewing has four white fasciae, whereas in *Gibbovalva magnoliae* forewing has a V-shaped speck at base and five white fasciae and in *Gibbovalva tricuneatella* forewing has three white fasciae which markedly dilate towards wing fold. In addition, *Gibbovalva clavata* resembles *Gibbovalva quadrifasciata* (Stainton) in the male genitalia, as in both species the ventral surface of valva is covered with lanceolate setae, but it is distinguishable by other characters.

Description. Adult (Fig. 4). Wingspan 7.0 mm. Head white, with frons fuscous. Labial palpus white, second segment apically and third segment basally with a fuscous spot on their outer side. Thorax white, its sides edged with fuscous line; tegula fuscous with white apex. Basal 1/3 of forewing white, with four black specks along costa, of which the last one smallest; distal 2/3 of forewing ochreous yellow with fuscous band along costa and four white, nearly equally spaced fasciae which obliquely extend outwards from costa to dorsum; two basal fasciae, approximately twice the width of the two distal ones, enclose a black spot on costa. Cilia black from dorsal third fascia to costal fourth fascia, white at the apical angle, the remaining cilia pale grey. Hind wing and its cilia pale grey. Legs with coxae and tarsi white; tarsi with three fuscous rings, the last tarsomere ochreous yellow apically. Profemur fuscous; protibia white in basal 1/3, the remaining part fuscous. Mesofemur with external surface fuscous, internal surface ochreous white; mesotibia white, with three fuscous rings, of which one at the basal 1/3, two at the distal part. Metafemur white, external side with a fuscous spot at base and middle respectively; metatibia white, with a median fuscous ring, the last tarsomere fuscous apically.

Male genitalia (Fig. 8). Tegumen approximately 400 μm long, tongue-like, slightly wider on apical half, densely covered with fine setae on ventral and dorsal surfaces and with a sparse row of longer setae on each side. Valva approx. 600 μm long, blade-shaped, slightly narrowed at base, obliquely truncated at apex and almost parallel-sided; costa straight with a clavate process at the basal 1/6, dorsum slightly upcurved near apex; inner surface covered with usual setae except for lanceolate setae clustered on distal part. Saccus thumb-shaped, rounded apically. Aedeagus nearly 650 μm long, tapering to a pointed apex from around the distal 1/4; vesica with acute, thorn-like cornuti arranged in rows from basal 1/3 to aedeagus subapex, some cornuti arranged between apical 1/5 and apex being larger than others. Antero-dorsal apodeme of the eighth tergite approx. 150 μm long, with slender sclerotization extending caudad to the middle of the eighth tergite; eighth sternite with a pair of very slender invaginations, nearly equal in length to dorsal apodeme.

Female. Unknown.

#### Type material.

Holotype ♂. China, Jiulian Mountain, Longnan, Jiangxi Province, 600 m, 30 March 2012, leg. Jiasheng Xu; genitalia slide No. B12020, in ICCC.

#### Etymology.

The species name is derived from the Latin “*clavatus*”, meaning “clavate”, in reference to the costal process of valva.

#### Distribution.

China (Jiangxi).

## Supplementary Material

XML Treatment for Eumetriochroa

XML Treatment for Eumetriochroa hiranoi

XML Treatment for Eumetriochroa hederae

XML Treatment for Metriochroa

XML Treatment for Metriochroa alboannulata

XML Treatment for Gibbovalva

XML Treatment for Gibbovalva clavata
